# Anti-Aggregative Effect of the Antioxidant DJ-1 on the TPPP/p25-Derived Pathological Associations of Alpha-Synuclein

**DOI:** 10.3390/cells10112909

**Published:** 2021-10-27

**Authors:** Judit Oláh, Attila Lehotzky, Tibor Szénási, Judit Ovádi

**Affiliations:** Cell Architecture Lab, Research Center for Natural Sciences, Institute of Enzymology, 1117 Budapest, Hungary; lehotzky.attila@ttk.hu (A.L.); szenasi.tibor@ttk.hu (T.S.)

**Keywords:** alpha-synuclein, TPPP/p25, DJ-1, parkinsonism, anti-aggregative effect, BiFC

## Abstract

DJ-1, a multi-functional protein with antioxidant properties, protects dopaminergic neurons against Parkinson’s disease (PD). The oligomerization/assembly of alpha-synuclein (SYN), promoted by Tubulin Polymerization Promoting Protein (TPPP/p25), is fatal in the early stage of PD. The pathological assembly of SYN with TPPP/p25 inhibits their proteolytic degradation. In this work, we identified DJ-1 as a new interactive partner of TPPP/p25, and revealed its influence on the association of TPPP/p25 with SYN. DJ-1 did not affect the TPPP/p25-derived tubulin polymerization; however, it did impede the toxic assembly of TPPP/p25 with SYN. The interaction of DJ-1 with TPPP/p25 was visualized in living human cells by fluorescence confocal microscopy coupled with Bifunctional Fluorescence Complementation (BiFC). While the transfected DJ-1 displayed homogeneous intracellular distribution, the TPPP/p25-DJ-1 complex was aligned along the microtubule network. The anti-aggregative effect of DJ-1 on the pathological TPPP/p25-SYN assemblies was established by the decrease in the intensity of their intracellular fluorescence (BiFC signal) and the increase in the proteolytic degradation of SYN complexed with TPPP/p25 due to the DJ-1-derived disassembly of SYN with TPPP/p25. These data obtained with HeLa and SH-SY5Y cells revealed the protective effect of DJ-1 against toxic SYN assemblies, which assigns a new function to the antioxidant sensor DJ-1.

## 1. Introduction

Parkinson’s disease (PD), the second most common neurodegenerative disorder, is characterized by the loss of dopaminergic neurons [1,2], the histopathological hallmark of which are the Lewy bodies [3]. One of the key pathological hallmarks of PD is aggregation of the intrinsically disordered alpha-synuclein (SYN). SYN is expressed in pre-synapses under physiological conditions; its expression is regulated in a neuronal cell type-dependent manner [4]. SYN has been shown to be involved in neurotransmitter release, synaptic vesicle pool maintenance and dopamine metabolism [2,5]. Under physiological conditions, the chameleon monomeric SYN exists in distinct conformational states [6,7]; the formation of the oligomers and β-sheet-rich fibrillar aggregates of the protein are characteristic of the pathological conditions, and these aggregated and fibrillary forms are accumulated in pathological inclusions [8]. The small, soluble oligomeric forms are now considered the most toxic species formed at the early stage of the disease [9,10].

Tubulin Polymerization Promoting Protein (TPPP/p25), similarly to SYN, is a chameleon protein and a hallmark of PD as well, which is co-enriched and co-localized with SYN in Lewy bodies [11]. TPPP/p25, as a Microtubule Associated Protein (MAP), regulates the dynamics and stability of the microtubule network by its microtubule bundling and acetylation enhancing activities [12,13,14]. At pathological conditions, TPPP/p25 induces the oligomerization/aggregation of SYN [11,15]. 

DJ-1 is a folded, highly conserved protein expressed ubiquitously, including in the brain [16,17]. It displays a plethora of functions, such as an antioxidative stress reaction, enzymatic and chaperone activities as well as transcriptional regulation [18,19]. The Cys oxidation plays roles in the function and dysfunction of DJ-1 [20]. This oxidative modification at the conserved Cys_106_ residue acts as a sensor of cellular redox homeostasis and participates in cytoprotective signaling pathways [21]. DJ-1 has been shown to interact with monomeric and oligomeric SYN in living cells; its overexpression reduces SYN aggregation [22,23,24]. A strong relationship between PD and DJ-1 has been documented, and mutations in DJ-1 results in the early onset of familial PD [25,26]. DJ-1 deficiency leads to increased oxidative stress as well as decreased SYN degradation, and these factors could all contribute to SYN aggregation [17]. 

In this work, we identified DJ-1 as a new binding partner of TPPP/p25, and revealed its modulating effect on the pathological association of SYN and TPPP/p25 using human recombinant proteins and living human cell models.

## 2. Materials and Methods

### 2.1. Antibodies

The used antibodies can be found in Table 1.

### 2.2. Plasmid Constructs 

Bacterial expression plasmids containing the insert for human TPPP/p25 and SYN were prepared and purified as described previously [14,27].

mVenus-DJ-1 and mCherry-DJ-1, corresponding to the full-length protein, were amplified by PCR using the forward primer 5′-GCGACCTCGAGTCATGGCTTCCAAAAGAGCTCTGGTCATCC-3′ and reverse primer 5′-CAGCCGGATCCCTAGTCTTTAAGAACAAGTGG-3′. After digestion with XhoI and BamHI restriction enzymes, inserts were ligated into the mVenusC1 and mCherryC1 vector. 

For the Bimolecular Fluorescence Complementation (BiFC) experiments, the V^N^-SYN and V^C^-SYN containing the insert for human full-length SYN were prepared as described previously [28]. The V^C^-TPPP/p25 construct containing the insert for human full-length TPPP/p25 was prepared as described previously [29]. To insert the DJ-1 cDNA in either the pBiFC-V^N1–173^ or pBiFC-V^C155–238^, the following primers were used for PCR amplification: 5-GCGACCTCGAGTCATGGCTTCCAAAAGAGCTCTGGTCATCC-3 (forward) and 5-CAGCCGGATCCCTAGTCTTTAAGAACAAGTGG-3 (reverse). The PCR product was purified and digested with XhoI-BamHI restriction enzymes, then inserted into the XhoI and BamHI sites in the BiFC vectors. 

Restriction mapping and sequencing were used to verify the sequences of all constructs.

### 2.3. Expression and Purification of Proteins 

The C-terminal hexaHis fusion human recombinant TPPP/p25 was expressed in E. coli BL21 (DE3) and isolated on a HIS-Select™ Cartridge (Merck P6611) as described previously [28,30]. The human recombinant SYN was prepared as described previously [31]. Tubulin was purified from bovine brain [32]. 

The concentrations of the human recombinant proteins and tubulin were established by their absorbance at 280 nm with the following extinction coefficients (http://web.expasy.org/protparam, accessed on 15 February 2021): 10,095 M^−1^*cm^−1^ for TPPP/p25; 5960 M^−1^*cm^−1^ for SYN; and 50,310 M^−1^*cm^−1^ for tubulin. In turn, the protein concentration in the cellular extract was measured by the Bradford method [33].

### 2.4. Enzyme-Linked Immunosorbent Assay (ELISA) Experiments

Briefly, the plate was coated with 5 μg/mL DJ-1 overnight in phosphate-buffered saline (PBS), and the wells were blocked with 1 mg/mL bovine serum albumin in PBS for 1 h at room temperature. Then TPPP/p25 at a serial dilution was added, and its binding was quantified by a specific polyclonal TPPP/p25 antibody followed by addition of the corresponding secondary antibody conjugated to horseradish peroxidase, respectively (Table 1). In the case of the competitive ELISA, 500 nM SYN pre-incubated for 30 min without or with 1.25 μM or 2.5 μM TPPP/p25 was added to the immobilized DJ-1. The bound SYN was quantified by a specific SYN antibody as described above.

For the quantification of the immunocomplex, o-phenylenediamine with hydrogen peroxide as a substrate was applied, and the absorbance was read at 490 nm with an EnSpire Multimode Reader (Perkin Elmer, Waltham, MA, USA). The background value (no added proteins) was subtracted. The apparent binding constant (K_d_) was determined from the saturation curves by non-linear curve fitting (single binding site hyperbola model) using Origin 2018 64-bit software (Northampton, MA, USA).

### 2.5. Turbidity Measurements and Pelleting Experiments

The tubulin polymerization was visualized by turbidity measurements at 6 μM con-centration in the following buffer: 50 mM 2-(N-morpholino)ethanesulfonic acid buffer pH 6.6 containing 100 mM KCl, 1 mM dithioerythritol, 1 mM MgCl_2_ and 1 mM ethylene glycol tetraacetic acid at 37 °C with or without 10 μM DJ-1 and/or 10 μM SYN. The tubulin assembly was promoted by 3 μM of TPPP/p25. The absorbance was detected at 350 nm by a Cary 100 spectrophotometer (Varian, Mulgrave, Australia). In the case of the pelleting experiment, the samples at the end of the turbidity measurements were centrifuged (17,000× *g* for 15 min at 37 °C). The resuspended pellet and supernatant fractions were analyzed by sodium dodecyl sulfate polyacrylamide gel (16%) electrophoresis (SDS-PAGE); the intensity of the bands was determined by densitometry using ImageJ 1.49. 

### 2.6. Cell Culture, Transfection and Manipulation

HeLa cells (ATCC^®^ CCL-2™) were grown in Dulbecco’s Modified Eagle’s Medium (high glucose) supplemented with 10% (*v*/*v*) fetal bovine serum (FBS), 100 μg/mL kanamycin and Antibiotic Antimycotic Solution (all from Merck; complete medium) in a humidified incubator at 37 °C with 5% CO_2_. 

Neuroblastoma SH-SY5Y cells (ATCC CRL-2266) were cultured in DME/F12 medium, supplemented with 10% FBS, 100 μg/mL kanamycin and Antibiotic Antimycotic Solution (all Merck; complete medium). Cells were grown in a humidified incubator at 37 °C, in a 5% CO_2_ atmosphere. The line was used between 10 and 30 passages for the experiments.

For cellular experiments, 5 × 10^4^ HeLa or SH-SY5Y cells (in 24-well plates) were transfected with DJ-1-expressing plasmid using the Turbofect (Thermo Fisher Scientific) transfection reagent according to the manufacture’s protocol. As the control, non-transfected cells were used as described previously [34]. The human recombinant SYN and/or TPPP/p25 were taken up from the medium, and then the cells were incubated for a further 4 h. The concentrations of TPPP/p25 and SYN were 80 nM and 800 nM, respectively. 

The visualization of the hetero-associations of the proteins was archived by detecting the BiFC signal originated from transfections of coverslip-plated HeLa cells with the mVenus BiFC constructs of the proteins, as described previously [28,34]. Nuclei were counterstained with Hoescht 33342. The BiFC signal was detected on formaldehyde-fixed (4% in PBS) samples by confocal microscopy. To visualize the microtubule structures and the BiFC signal by microscopy, the samples were fixed by cold methanol. The samples were then processed for tubulin immunocytochemistry and the fluorescent constructs were detected directly.

For Western blot, the HeLa or SH-SY5Y cells were washed with PBS, and then lysed in situ in a 1x reducing sample buffer.

### 2.7. Western Blot

The samples (equal amount of proteins loaded) were separated by 13.5% SDS-PAGE and were electrotransferred onto Immobilon-PSQ transfer membranes. Then the membranes were treated with 2% paraformaldehyde in PBS containing 0.1% Tween-20 for 30 min and washed with PBS supplemented with 0.1% Tween-20 before blocking [35]. For the visualization and quantification of the protein bands, specific antibodies were used as follows: a rabbit polyclonal GFP antibody (for mVenus-DJ-1), a rabbit polyclonal SYN antibody, a rat polyclonal TPPP/p25 antibody and a mouse monoclonal beta-actin antibody, sequentially (Table 1). Antibody binding was visualized by the appropriate IgG-peroxidase conjugate by a Bio-Rad ChemiDoc MP Imaging system (Bio-Rad, Hercules, CA, USA) using Immobilon Western substrate (Merck), as described previously [34]. For densitometric analysis, the intensity of the bands was analyzed by ImageJ 1.49. The data were normalized on the basis of the loading control (beta-actin). 

### 2.8. Immunofluorescence Microscopy 

Confocal images were acquired with a Zeiss LSM 710 microscope (Zeiss, Jena, Germany) controlled by the LSM Zen 2010 B SP1 software using oiled 40 × NA = 1.4 Plan Apo objectives and the following lasers: Diode laser at 405 nm for Hoescht 33342, Argon laser at 514 nm for mVenus or BiFC and HeNe laser at 543 nm for Alexa 546 and mCherry-tag; the signals were acquired one by one.

Tiff formats of the images were created from the original pictures (2048 × 2048 pixels, 72 dpi, 8-bit lsm images). Complex images were created with Adobe Photoshop 2021 CC (San José, CA, USA). The BiFC intensity per cell was counted for each sample by an observer blinded to the experimental conditions. For densitometric analysis, the images were collected under constant exposure parameters, then the mVenus signal intensity was determined by ImageJ. The whole territory of the cells was outlined using the Freehand Line tool; then, the sum of the grey values of the pixels in the selection (integrated density) was taken and the background was subtracted.

For the co-localization study of the green (BiFC or mVenus signal) and the red (tubulin) channels, images were analyzed using the Co-localization panel of the LSM Zen 2010 B SP1 software. The values of the Pearson’s coefficient (R) and the mean intensity were determined by the LSM Zen 2010 B SP1 software.

### 2.9. Statistical Analysis

The values are presented as the mean ± SD (standard deviation) of at least 3 independent experiments, or a box diagram is shown. The Shapiro–Wilk test was used to test whether the data was significantly drawn from a normally distributed population (*p* < 0.05). Statistical comparisons were performed with one-way ANOVA followed by Tukey’s test for multiple comparisons or with a Kruskal–Wallis non-parametric test using Origin 2018 64-bit or GraphPad Prism 8.3.0 software (Northampton, MA, USA). Values were considered to be significant if the calculated p value was <0.05 (* *p* < 0.05, ** *p* < 0.01, ***p < 0.001 and **** *p* < 0.0001). 

## 3. Results and Discussion

### 3.1. TPPP/p25 Is a New Interacting Partner of DJ-1

**Figure 1:**
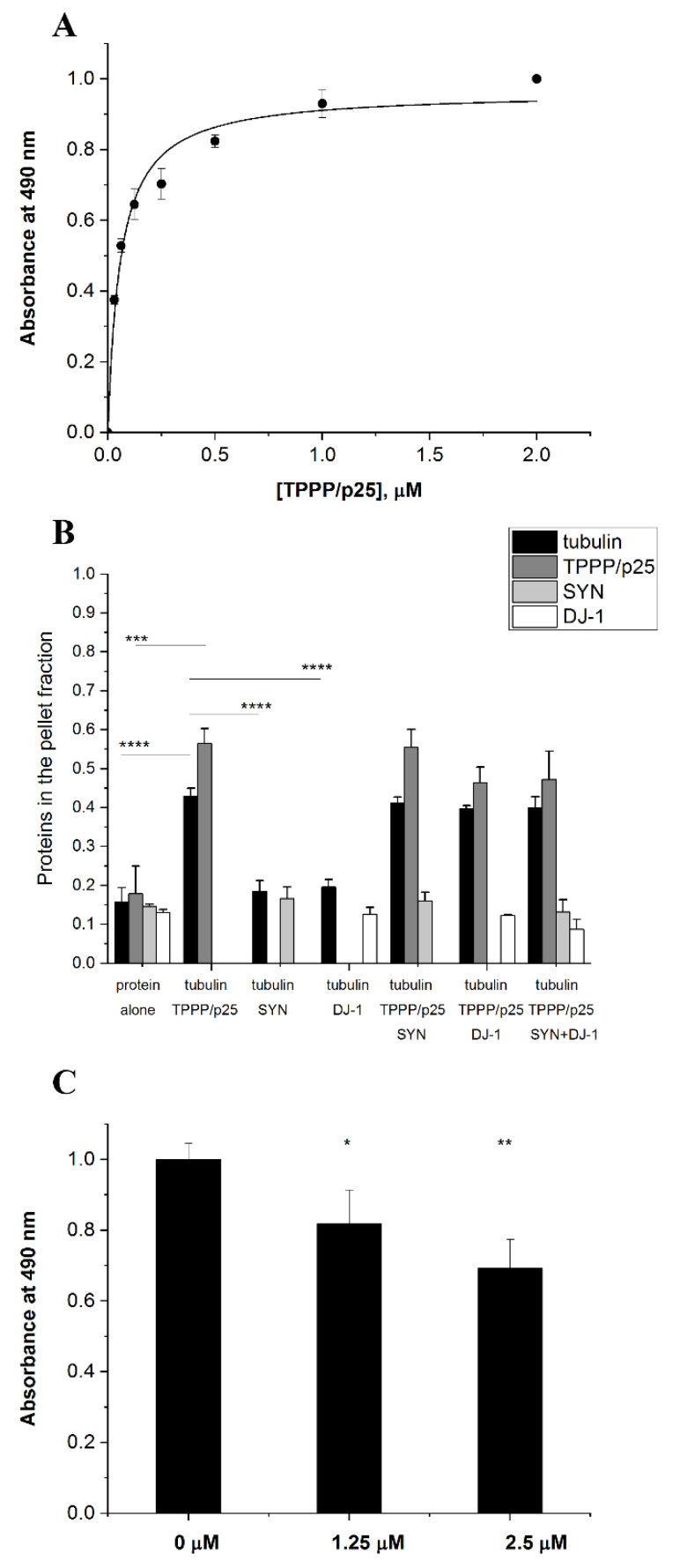
TPPP/p25 is an interacting partner of DJ-1. (**A**) Binding of TPPP/p25 to immobilized DJ-1 detected in an ELISA experiment. The K_d_ was evaluated from the saturation curve by non-linear curve fitting (K_d_ 58.1 ± 9.49 nM). (**B**) Effect of DJ-1 on the tubulin polymerization-promoting potency of TPPP/p25 using the pelleting experiment, as described in the Material and Methods. The partition of the proteins in the pellet (P) fraction was quantified by densitometric analysis (n = 3–4). (**C**) Inhibitory effect of TPPP/p25 on the interaction of -SYN with DJ-1 obtained by a competitive ELISA experiment. Data were normalized with respect to SYN without TPPP/p25 (n = 4). (**A**–**C**) Data are presented as the mean ± SD. Statistical comparisons were performed with one-way ANOVA followed by Tukey’s test, as compared to the control or as the lines indicate (* *p* < 0.05, ** *p* < 0.01, ***p < 0.001 and **** *p* < 0.0001).

SYN and TPPP/p25, hallmark proteins of PD, form pathological assemblies, including their co-enrichment and co-localization in post-mortem human brain [11]. The interaction of DJ-1 with SYN and its effect against the formation of toxic SYN oligomers/aggregates has been documented [23,24]. Now we characterized the association of DJ-1 with TPPP/p25, a pathological partner of SYN. ELISA experiments using human recombinant proteins were performed as described in detail in the Materials and Methods; TPPP/p25 was added at various concentrations to DJ-1 immobilized on the plate, and the interaction was detected by a TPPP/p25-specific antibody. Figure 1A shows that TPPP/p25 firmly binds to DJ-1 (K_d_ = 58.1 ± 9.5 nM), comparably to the interaction of TPPP/p25 with SYN (K_d_ = 48.9 ± 6.7 nM) [28,36].

TPPP/p25 promotes the tubulin polymerization into intact-like, double-walled microtubules and aggregate, causing their bundling, which stabilizes the dynamic microtubule network, promoting resistance against anti-mitotic agents [12,37]. Then, the effect of DJ-1 on the physiological functions of the TPPP/p25-derived tubulin assemblies was tested by turbidity measurements. The typical time courses of the TPPP/p25-induced tubulin polymerization are shown in Figure S1A, which do not show an initial lag phase due to the formation of aggregated microtubules besides the intact-like ones, as demonstrated earlier by electron microscopic studies [37]. The additions of DJ-1 and/or SYN do not result in alteration in the time-dependent turbidity, indicating that neither DJ-1 nor the DJ-1-SYN complex modify the ability of TPPP/p25 to promote the oligomerization/assembly of tubulin subunits.

Next, the effect of DJ-1 and/or SYN on the partition of the soluble tubulin and polymerized microtubules induced by TPPP/p25 was studied in a pelleting experiment. The samples of the turbidity measurements were used as described in the Materials and Methods. Figure 1B and Figure S1 show the partition of the proteins in the pellet and supernatant fractions, respectively. These data (Figure S1B–D) revealed that both tubulin and TPPP/p25 alone appear predominantly in the supernatant fraction; however, the addition of TPPP/p25 to tubulin altered the partition of the two proteins, as demonstrated previously [30]. The levels of both DJ-1 and SYN in the pellet fractions are very low, similar to that measured in the presence of tubulin and/or TPPP/p25. This finding suggests that their presence does not influence significantly the association of tubulin with TPPP/p25.

These findings underline that neither DJ-1 nor its mixture with SYN modifies the TPPP/p25-induced self-assembly of tubulin, suggesting the lack of modification potency of DJ-1 concerning the physiological functions of TPPP/p25.

Next, we investigated the nature of the association of SYN with DJ-1 in the presence of TPPP/p25 using a competitive ELISA assay. DJ-1 was immobilized on the plate, and then SYN was added without or with TPPP/p25. The effect of TPPP/p25 on the binding of SYN with the immobilized DJ-1 was detected by a SYN antibody, as described in the Materials and Methods. As shown in Figure 1C, TPPP/p25 inhibited the SYN binding to the immobilized DJ-1, suggesting the competition of the two hallmark proteins for DJ-1 binding. These in vitro data obtained with human recombinant proteins are indicative of the possible anti-aggregative effect of DJ-1 against the pathological TPPP/p25-SYN association.

### 3.2. Intracellular Association and Localization of DJ-1 with SYN and TPPP/p25

**Figure 2:**
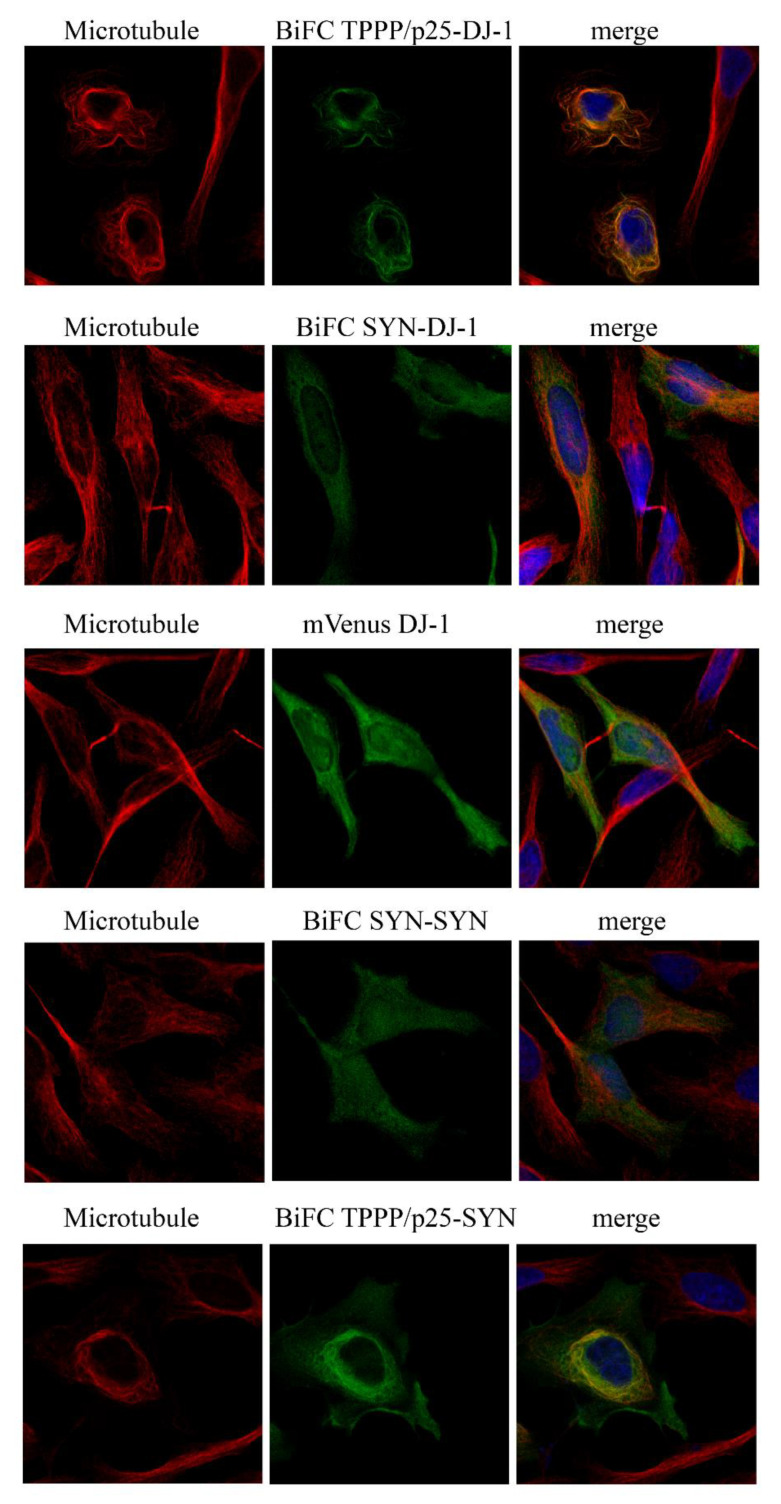
Hetero-association of DJ-1 with SYN or TPPP/p25 in living HeLa cells as visualized by BiFC technology (BiFC signal green). V^C^ and V^N^ fragments of Venus were conjugated to DJ-1 and TPPP/p25 (V^N^-DJ-1 and V^C^-TPPP/p25) or SYN (V^N^-SYN and V^C^-DJ-1), and their plasmids were co-transfected or mVenus-conjugated DJ-1 was transfected as the control. The associations of SYN with SYN or TPPP/p25 (V^N^-SYN and V^C^-TPPP/p25) visualized by BiFC technology are also shown. Alignment of the TPPP/p25-DJ-1 BiFC complex (green) on the microtubule network (red). All the details of the experiments are described in the Materials and Methods. Nuclei, blue (Hoescht 33342). Bar: 5 μm.

In order to validate the in vitro data at cellular level, the interactions of DJ-1 with SYN or TPPP/p25 were visualized in a living HeLa cell model by immunofluorescence microscopy coupled with BiFC technology. Venus fusion constructs of DJ-1 and SYN as well as DJ-1 and TPPP/p25 were produced by recombinant technology, as described in the Materials and Methods. The BiFC signal exploits of the intracellular interaction of the labeled proteins results in the close proximity of the two segments to the split Venus protein, producing a fluorescent emission (green BiFC signal). The empty Venus vectors virtually do not produce a BiFC signal (Figure S2). As shown in Figure 2, BiFC complexes are formed in living human cells by the co-expressions of DJ-1 with TPPP/p25 or SYN fused to the split Venus protein. 

In addition, our experiments performed with immunofluorescence confocal micros-copy showed that DJ-1 complexed with TPPP/p25, but not with SYN, is aligned along the microtubule network (Figure 2 and Table S1). Control experiment carried out with the full-length mVenus DJ-1 underlines its homogeneous intracellular distribution in the cyto-plasm (Figure 2), in agreement with the reported data [16,18]. The BiFC signal of the SYN–SYN complex does not show co-localization with the microtubule network, while TPPP/p25 complexed with SYN exhibits limited alignment along the microtubule network (Figure 2 and Table S1). However, the assembled/aggregated SYN-TPPP/p25 species has been visualized in the cytosol (Figure 2 and [27,30]), where DJ-1 may express its anti-aggregative effect.

### 3.3. Inhibitory Effect of DJ-1 on the Intracellular Association of TPPP/p25 to SYN

**Figure 3:**
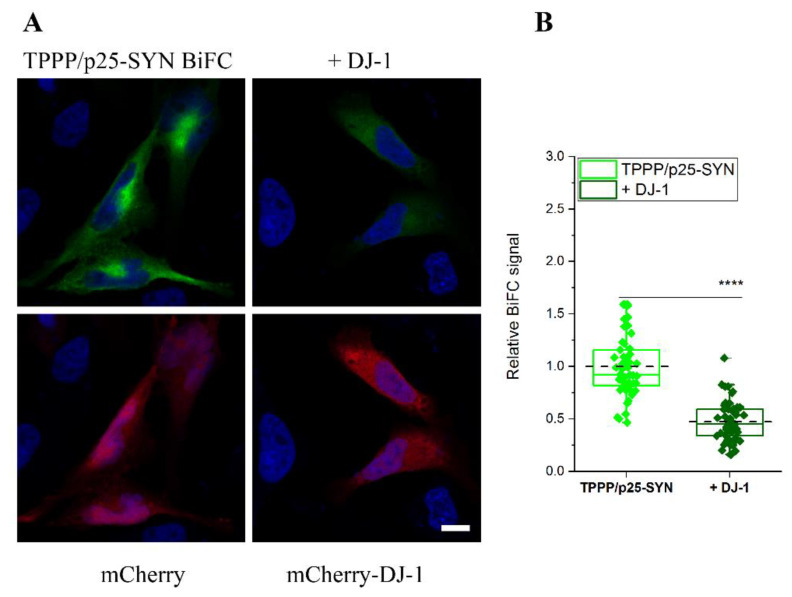
The effect of DJ-1 on the association of V^N^-SYN with V^C^-TPPP/p25 in a human cell line. (**A**) Representative images. V^C^- and V^N^-containing fragments of Venus were conjugated to SYN and TPPP/p25 sequences, respectively, and their plasmids were co-transfected into HeLa cells and with either mCherry (control cells) or mCherry-DJ-1 (mixture of 3 plasmids). Nuclei, blue (Hoescht 33342). Bar: 5 μm. (**B**) Quantification of the BiFC signal based on the individual cell fluorescence as described in the Material and Methods. Box extends from the 25th to 75th percentile with the middle solid green and the dashed black lines representing the median and the mean, respectively (n = 49 for TPPP/p25-SYN, n = 54 for + DJ-1). Statistical comparison was performed with one-way ANOVA followed by Tukey’s test (**** *p* < 0.0001).

After establishing the direct interaction of DJ-1 with both SYN and TPPP/p25, the two hallmark proteins of PD, the inhibitory potency of DJ-1 on their pathological association was visualized in living human cells by fluorescence confocal microscopy. Figure 3 illustrates that the co-transfected V^C^-TPPP/p25 and V^N^-SYN proteins form a BiFC complex according to our previously reported data [28]. The transfection of Venus-labelled proteins fused with SYN and TPPP/p25 as well as that of the mCherry-DJ-1 allowed to visualize the counteracting influence of DJ-1 on the assembly of SYN and TPPP/p25. Control experiments, in agreement with our previous data [29], underlined that the co-expressed empty Venus vectors virtually do not produce a BiFC signal either in the absence or presence of transfected mCherry-DJ-1 (Figure S2A,B).

Figure 3A shows representative images of the effect of DJ-1 on the BiFC signal of the pathological TPPP/p25-SYN complex (control cells were transfected with empty mCherry alone). The data obtained from these experiments were applied for the quantification of the influence of DJ-1 on the BiFC signal (Figure 3B). In a comparative study, the effect of DJ-1 on the SYN-SYN assembly was visualized (Figure S2C). The co-expressed empty Venus vectors virtually do not produce a BiFC signal in this experimental setup either (Figure S2D). The results underlined that DJ-1 reduced the SYN–SYN association, in agreement with the previously reported data [23], and the inhibitory potencies of DJ-1 on the homo- and hetero-associations of SYN are comparable under similar experimental conditions.

### 3.4. Effect of DJ-1 on the TPPP/p25-Inhibited Proteolytic Degradation of SYN Assembly

TPPP/p25 and SYN are co-enriched and co-localized in the inclusion bodies of the post-mortem brain tissue of patients with parkinsonism due to their cell-to-cell transmission [11,38]. Recently, we have revealed that the excess SYN and TPPP/p25 can be degraded by autophagy and/or ubiquitin–proteasome system, and their eliminations are inhibited in the presence of specific inhibitors, such as chloroquine and MG132, respectively, indicating their intracellular degradation [34]. In addition, we have reported that the hetero-association of the two hallmark proteins counteract their degradation [34].

In this set of experiments, the effect of DJ-1 on the degradation of the excess SYN complexed with TPPP/p25 was detected and quantified. SYN and/or TPPP/p25 were taken up from the medium by human cells to mimic the pathological circumstances (cell-to-cell transmission) [34]. Now, both HeLa and SH-SY5Y cells were used as cell models to monitor the anti-aggregative effect of DJ-1. The effect manifests itself in the enhanced degradation (reduced level) of SYN due to its release from its hetero-association with TPPP/p25. Accordingly, the levels of the complexed and uncomplexed SYN and TPPP/p25 were measured and quantified by Western blot.

**Figure 4:**
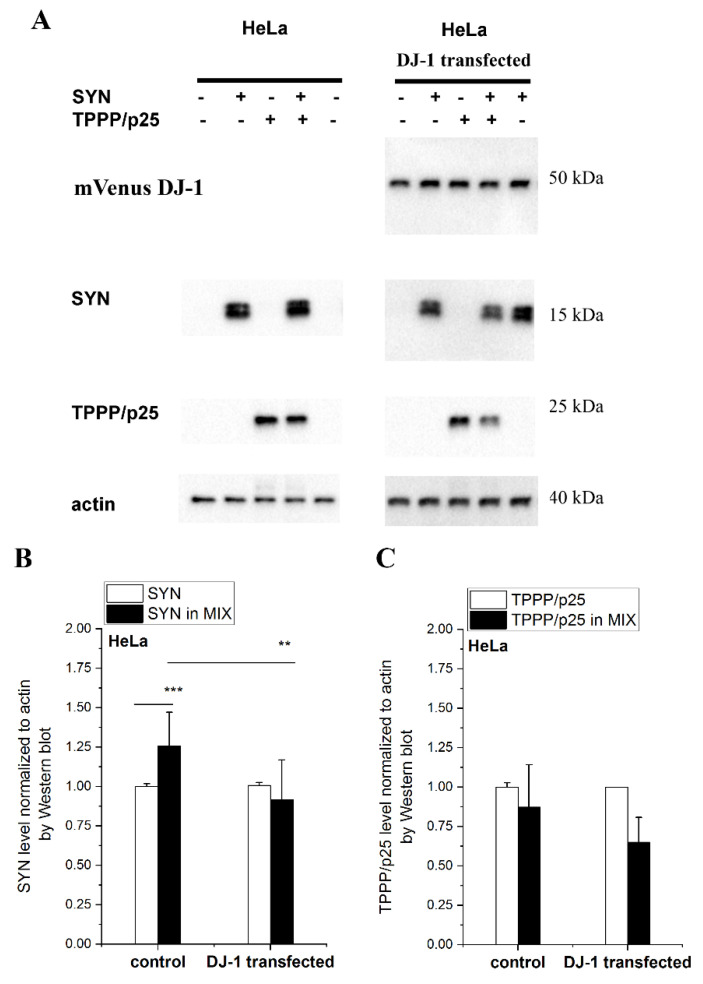
The effect of DJ-1 on the level of the degradation-resistant SYN and TPPP/p25. TPPP/p25 and/or SYN were taken up from the medium by the HeLa cells transfected with DJ-1. (**A**) Representative Western blots using cellular extracts without or with transfected DJ-1. The transfected mVenus-DJ-1 was quantified using GFP antibody. Actin as a loading control is also shown. (**B**,**C**) Proteins levels of SYN (**B**) and TPPP/p25 (**C**) were quantified by Western blot, as described in the Material and Methods. Data are normalized with respect to the added hallmark protein alone (SYN or TPPP/p25), and presented as the mean ± SD of at least 3 independent experiments. Statistical comparisons were performed with a Kruskal–Wallis non-parametric test (*** *p* < 0.001 and ** *p* < 0.01 compared to the control or as the lines indicate).

Figure 4 shows a representative set of experiments carried out with HeLa cells. The SYN level is higher when it is complexed with TPPP/p25 as compared to SYN alone, as we have recently reported [34]; however, the addition of DJ-1 reduced the SYN level in the presence of TPPP/p25 (MIX) to the control level (no added TPPP/p25). These data suggest that DJ-1 is able to abolish the inhibitory effect of TPPP/p25 on SYN degradation by its anti-aggregative potency exerted on the pathological interaction of the PD hallmarks. A similar result was obtained when this set of experiments was carried out with SH-SY5Y cells (Figure S3), indicating that the inhibitory potency of DJ-1 is achieved in neuronal cells as well.

Considering our findings obtained in human living cells, these results are in line with the reported data that DJ-1 deficiency increased the accumulation and aggregation of SYN in both SH-SY5Y cells and PD animal models, while its overexpression reduced the SYN levels [17].

## 4. Concluding Remarks

SYN and TPPP/p25 display multiple functions, including physiological and pathological ones; in addition, they have high conformational plasticity, and consequently these proteins are challenging drug targets [39]. Recently, we have proposed an innovative strategy: targeting the interface between TPPP/p25-SYN in this pathological complex without affecting the physiological functions of these proteins [27,28,30]. To achieve this, the use of fragments of the partner proteins or drug-like agents, such as peptidomimetic foldamers, has been proposed [40].

DJ-1 can inhibit both the oligomerization of SYN and the hetero-association of SYN with TPPP/p25; this hetero-association initiates formation of highly toxic species in PD. The observation that DJ-1 promotes the degradation of TPPP/p25-derived SYN assemblies may ensure therapeutic potency. These results indicate that DJ-1 is a sensitive modulator of the initial steps of the aggregation process both by binding directly to the proteins and by influencing the degradative pathways. This function of DJ-1 is likely to modulate the interface between SYN and TPPP/p25 by either direct competition or by indirectly inducing conformation changes at the interface of the pathological complex. Hence, the peptide fragments affecting the association of SYN with TPPP/p25 may contribute to a precious lead for drug development.

## Figures and Tables

**Table 1:** Antibodies used in this work.

Antibody	Company	Catalog Number	Dilution
rat polyclonal anti-TPPP/p25	[11]		1:5000
rabbit polyclonal anti-SYN	Merck (Darmstadt, Germany)	S3062	1:5000
mouse monoclonal anti-tubulin	Merck	T9026	1:1000 ^1^
mouse monoclonal anti-beta-actin	Thermo Fisher Scientific (Waltham, MA, USA)	MA1-140	1:5000
rabbit polyclonal anti-GFP	Thermo Fisher Scientific	A-11122	1:2000
anti-rat IgG, HRP-linked	Merck	A9037	1:5000
anti-mouse IgG, HRP-linked	Merck	A2554	1:5000
anti-rabbit IgG, HRP-linked	Thermo Fisher Scientific	32260	1:5000
anti-mouse IgG, Alexa-546 linked	Thermo Fisher Scientific	A11003	1:1000 ^1^

^1^ Dilution for immunofluorescence microscopy.

## Data Availability

All primary data can be found in Data_for_figures.xlsx Excel file.

## Supplementary Materials

The following are available online at https://www.mdpi.com/article/10.3390/cells10112909/s1, Figure S1: TPPP/p25-induced tubulin polymerization. Figure S2: The dynamic association of VN-SYN with VC-TPPP/p25 as visualized by BiFC technology. Figure S3: The effect of DJ-1 on the level of the degradation-resistant hallmark proteins in SH-SY5Y cells. Table S1: Quantitative analyses of pixel intensities and co-localization analysis (Pearson’s correlation coefficient; R) of the green (BiFC or mVenus signal) and the red (tubulin) channels.

## References

[1] Kalia L.V., Lang A.E. (2015). Parkinson’s disease. Lancet.

[2] Mochizuki H., Choong C.J., Masliah E. (2018). A refined concept: Alpha-synuclein dysregulation disease. Neurochem. Int..

[3] Spillantini M.G., Schmidt M.L., Lee V.M., Trojanowski J.Q., Jakes R., Goedert M. (1997). Alpha-synuclein in Lewy bodies. Nature.

[4] Taguchi K., Watanabe Y., Tsujimura A., Tanaka M. (2019). Expression of alpha-synuclein is regulated in a neuronal cell type-dependent manner. Anat. Sci. Int..

[5] Sulzer D., Edwards R.H. (2019). The physiological role of alpha-synuclein and its relationship to Parkinson’s Disease. J. Neurochem..

[6] Uversky V.N. (2003). A protein-chameleon: Conformational plasticity of alpha-synuclein, a disordered protein involved in neurodegenerative disorders. J. Biomol. Struct. Dyn..

[7] Frey B., AlOkda A., Jackson M.P., Riguet N., Duce J.A., Lashuel H.A. (2021). Monitoring alpha-synuclein oligomerization and aggregation using bimolecular fluorescence complementation assays: What you see is not always what you get. J. Neurochem..

[8] Goedert M., Jakes R., Spillantini M.G. (2017). The Synucleinopathies: Twenty Years On. J. Parkinsons Dis..

[9] Ono K. (2017). The Oligomer Hypothesis in alpha-Synucleinopathy. Neurochem. Res..

[10] Surguchev A.A., Surguchov A. (2017). Synucleins and Gene Expression: Ramblers in a Crowd or Cops Regulating Traffic?. Front. Mol. Neurosci..

[11] Kovacs G.G., Laszlo L., Kovacs J., Jensen P.H., Lindersson E., Botond G., Molnar T., Perczel A., Hudecz F., Mezo G. (2004). Natively unfolded tubulin polymerization promoting protein TPPP/p25 is a common marker of alpha-synucleinopathies. Neurobiol. Dis..

[12] Lehotzky A., Tirián L., Tőkési N., Lénárt P., Szabó B., Kovács J., Ovádi J. (2004). Dynamic targeting of microtubules by TPPP/p25 affects cell survival. J. Cell. Sci..

[13] Lehotzky A., Lau P., Tokesi N., Muja N., Hudson L.D., Ovadi J. (2010). Tubulin polymerization-promoting protein (TPPP/p25) is critical for oligodendrocyte differentiation. Glia.

[14] Tőkési N., Lehotzky A., Horvath I., Szabo B., Olah J., Lau P., Ovadi J. (2010). TPPP/p25 promotes tubulin acetylation by inhibiting histone deacetylase 6. J. Biol. Chem..

[15] Lindersson E., Lundvig D., Petersen C., Madsen P., Nyengaard J.R., Hojrup P., Moos T., Otzen D., Gai W.P., Blumbergs P.C. (2005). p25alpha Stimulates alpha-synuclein aggregation and is co-localized with aggregated alpha-synuclein in alpha-synucleinopathies. J. Biol. Chem..

[16] van der Vlag M., Havekes R., Heckman P.R.A. (2020). The contribution of Parkin, PINK1 and DJ-1 genes to selective neuronal degeneration in Parkinson’s disease. Eur. J. Neurol..

[17] Xu C.Y., Kang W.Y., Chen Y.M., Jiang T.F., Zhang J., Zhang L.N., Ding J.Q., Liu J., Chen S.D. (2017). DJ-1 Inhibits alpha-Synuclein Aggregation by Regulating Chaperone-Mediated Autophagy. Front. Aging Neurosci..

[18] Ariga H., Takahashi-Niki K., Kato I., Maita H., Niki T., Iguchi-Ariga S.M. (2013). Neuroprotective function of DJ-1 in Parkinson’s disease. Oxid. Med. Cell..

[19] Dolgacheva L.P., Berezhnov A.V., Fedotova E.I., Zinchenko V.P., Abramov A.Y. (2019). Role of DJ-1 in the mechanism of pathogenesis of Parkinson’s disease. J. Bioenerg. Biomembr..

[20] Wilson M.A. (2011). The role of cysteine oxidation in DJ-1 function and dysfunction. Antioxid. Redox Signal..

[21] Bahmed K., Boukhenouna S., Karim L., Andrews T., Lin J., Powers R., Wilson M.A., Lin C.R., Messier E., Reisdorph N. (2019). The effect of cysteine oxidation on DJ-1 cytoprotective function in human alveolar type II cells. Cell Death Dis..

[22] Shendelman S., Jonason A., Martinat C., Leete T., Abeliovich A. (2004). DJ-1 is a redox-dependent molecular chaperone that inhibits alpha-synuclein aggregate formation. PLoS Biol..

[23] Zondler L., Miller-Fleming L., Repici M., Goncalves S., Tenreiro S., Rosado-Ramos R., Betzer C., Straatman K.R., Jensen P.H., Giorgini F. (2014). DJ-1 interactions with alpha-synuclein attenuate aggregation and cellular toxicity in models of Parkinson’s disease. Cell Death Dis..

[24] Kumar R., Kumar S., Hanpude P., Singh A.K., Johari T., Majumder S., Maiti T.K. (2019). Partially oxidized DJ-1 inhibits alpha-synuclein nucleation and remodels mature alpha-synuclein fibrils in vitro. Commun. Biol..

[25] Bonifati V., Rizzu P., van Baren M.J., Schaap O., Breedveld G.J., Krieger E., Dekker M.C., Squitieri F., Ibanez P., Joosse M. (2003). Mutations in the DJ-1 gene associated with autosomal recessive early-onset parkinsonism. Science.

[26] Klein C., Westenberger A. (2012). Genetics of Parkinson’s disease. Cold Spring Harb. Perspect. Med..

[27] Szunyogh S., Olah J., Szenasi T., Szabo A., Ovadi J. (2015). Targeting the interface of the pathological complex of alpha-synuclein and TPPP/p25. Biochim. Biophys. Acta.

[28] Szenasi T., Olah J., Szabo A., Szunyogh S., Lang A., Perczel A., Lehotzky A., Uversky V.N., Ovadi J. (2017). Challenging drug target for Parkinson’s disease: Pathological complex of the chameleon TPPP/p25 and alpha-synuclein proteins. Biochim. Biophys. Acta Mol. Basis Dis..

[29] Olah J., Szenasi T., Szunyogh S., Szabo A., Lehotzky A., Ovadi J. (2017). Further evidence for microtubule-independent dimerization of TPPP/p25. Sci. Rep..

[30] Tokesi N., Olah J., Hlavanda E., Szunyogh S., Szabo A., Babos F., Magyar A., Lehotzky A., Vass E., Ovadi J. (2014). Identification of motives mediating alternative functions of the neomorphic moonlighting TPPP/p25. Biochim. Biophys. Acta.

[31] Paik S.R., Lee J.H., Kim D.H., Chang C.S., Kim J. (1997). Aluminum-induced structural alterations of the precursor of the non-A beta component of Alzheimer’s disease amyloid. Arch. Biochem. Biophys..

[32] Na C.N., Timasheff S.N. (1986). Interaction of vinblastine with calf brain tubulin: Multiple equilibria. Biochemistry.

[33] Bradford M.M. (1976). A rapid and sensitive method for the quantitation of microgram quantities of protein utilizing the principle of protein-dye binding. Anal. Biochem..

[34] Lehotzky A., Olah J., Fekete J.T., Szenasi T., Szabo E., Gyorffy B., Varady G., Ovadi J. (2021). Co-Transmission of Alpha-Synuclein and TPPP/p25 Inhibits Their Proteolytic Degradation in Human Cell Models. Front. Mol. Biosci..

[35] Sasaki A., Arawaka S., Sato H., Kato T. (2015). Sensitive western blotting for detection of endogenous Ser129-phosphorylated alpha-synuclein in intracellular and extracellular spaces. Sci. Rep..

[36] Olah J., Vincze O., Virok D., Simon D., Bozso Z., Tokesi N., Horvath I., Hlavanda E., Kovacs J., Magyar A. (2011). Interactions of pathological hallmark proteins: Tubulin polymerization promoting protein/p25, beta-amyloid, and alpha-synuclein. J. Biol. Chem..

[37] Hlavanda E., Kovacs J., Olah J., Orosz F., Medzihradszky K.F., Ovadi J. (2002). Brain-specific p25 protein binds to tubulin and microtubules and induces aberrant microtubule assemblies at substoichiometric concentrations. Biochemistry.

[38] Vargas J.Y., Grudina C., Zurzolo C. (2019). The prion-like spreading of alpha-synuclein: From in vitro to in vivo models of Parkinson’s disease. Ageing Res. Rev..

[39] Olah J., Ovadi J. (2019). Pharmacological targeting of alpha-synuclein and TPPP/p25 in Parkinson’s disease: Challenges and opportunities in a Nutshell. FEBS Lett..

[40] Olah J., Lehotzky A., Szunyogh S., Szenasi T., Orosz F., Ovadi J. (2020). Microtubule-Associated Proteins with Regulatory Functions by Day and Pathological Potency at Night. Cells.

