# Supplementary material for: Anti-Aggregative Effect of the Antioxidant DJ-1 on the TPPP/p25-Derived Pathological Associations of Alpha-Synuclein
Source: Cells. 2021 Oct 27;10(11):2909. doi: 10.3390/cells10112909 (PMC8616041; doi:10.3390/cells10112909)
Supplement: Supplementary file 1 [file cells-10-02909-s001.zip › Supplement_211022.pdf]

Supplementary Material

ANTI-AGGREGATIVE EFFECT OF THE ANTIOXIDANT DJ-1 ON THE TPPP/p25-DERIVED PATHOLOGICAL ASSOCIATIONS OF ALPHA-SYNUCLEIN

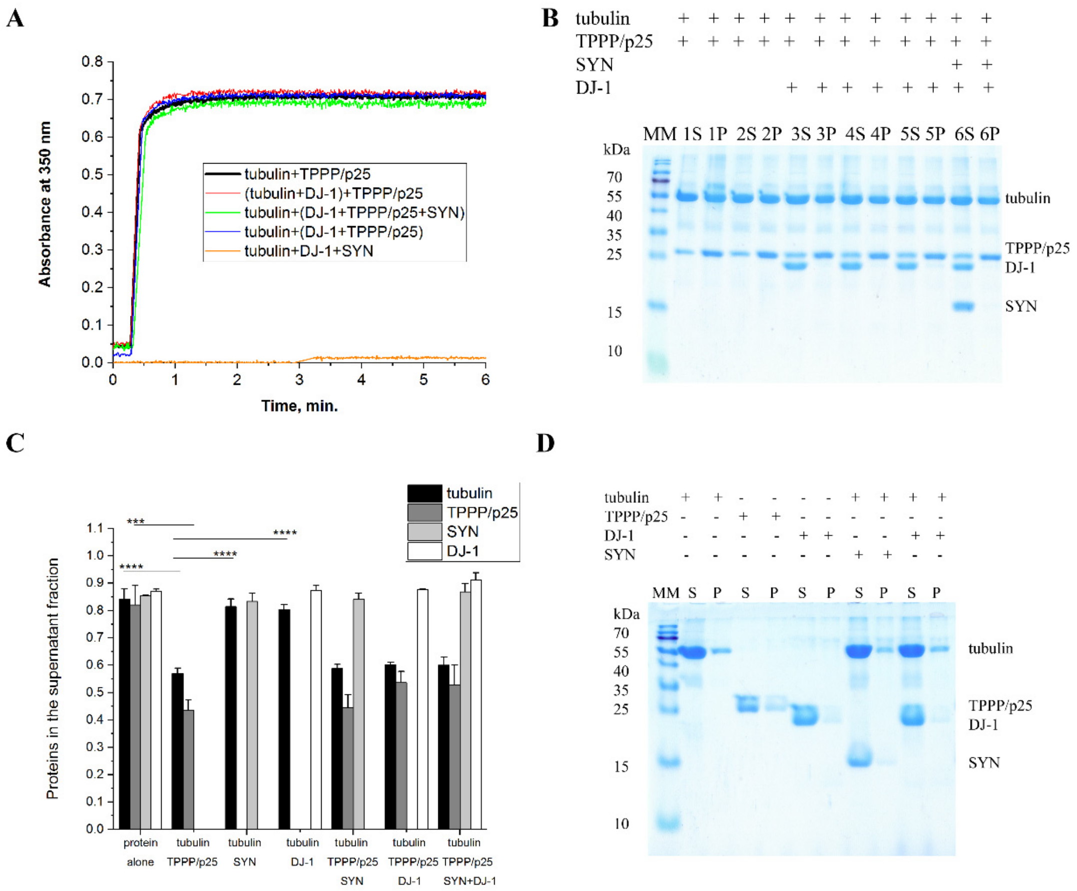

Figure S1. TPPP/p25-induced tubulin polymerization. (A) Turbidity measurement. (B) Pelleting experiment. Tubulin (6 μM) was incubated with TPPP/p25 (3 μM), DJ-1 (10 μM), and/or SYN (10 μM) at 37 °C in polymerization buffer. After the polymerization, the samples were centrifuged at 17,000 g for 15 min at 37 °C, the pellet (P) and the supernatant (S) fractions were separated, and were analyzed by 16% SDS-PAGE. Polymerization was induced by adding TPPP/p25 (sample 1 and 2) or (TPPP/p25 and DJ-1) (sample 5) or (TPPP/p25, SYN and DJ-1) (sample 6) to tubulin. In the cases of sample 3 and 4, TPPP/p25 was added to tubulin incubated with DJ-1. (C) Effect of DJ-1 on the tubulin polymerization-promoting potency of TPPP/p25 by pelleting experiment as described in the Material and Methods. The partition of the proteins in the supernatant (S) fraction was quantified by densitometric analysis (n=3-4). Data are presented as the mean ± SD. Statistical comparisons were performed with one-way ANOVA followed by Tukey's test, as compared to control or as the lines indicate. (D) Pelleting experiment. The proteins (tubulin 6 μM, TPPP/p25 3 μM, DJ-1 10 μM, SYN 10 μM) was incubated at 37 °C in polymerization buffer for 15 min. The samples were centrifuged at 17,000 g for 15 min at 37 °C, the pellet (P) and the supernatant (S) fractions were separated, and were analyzed by 16% SDS-PAGE.

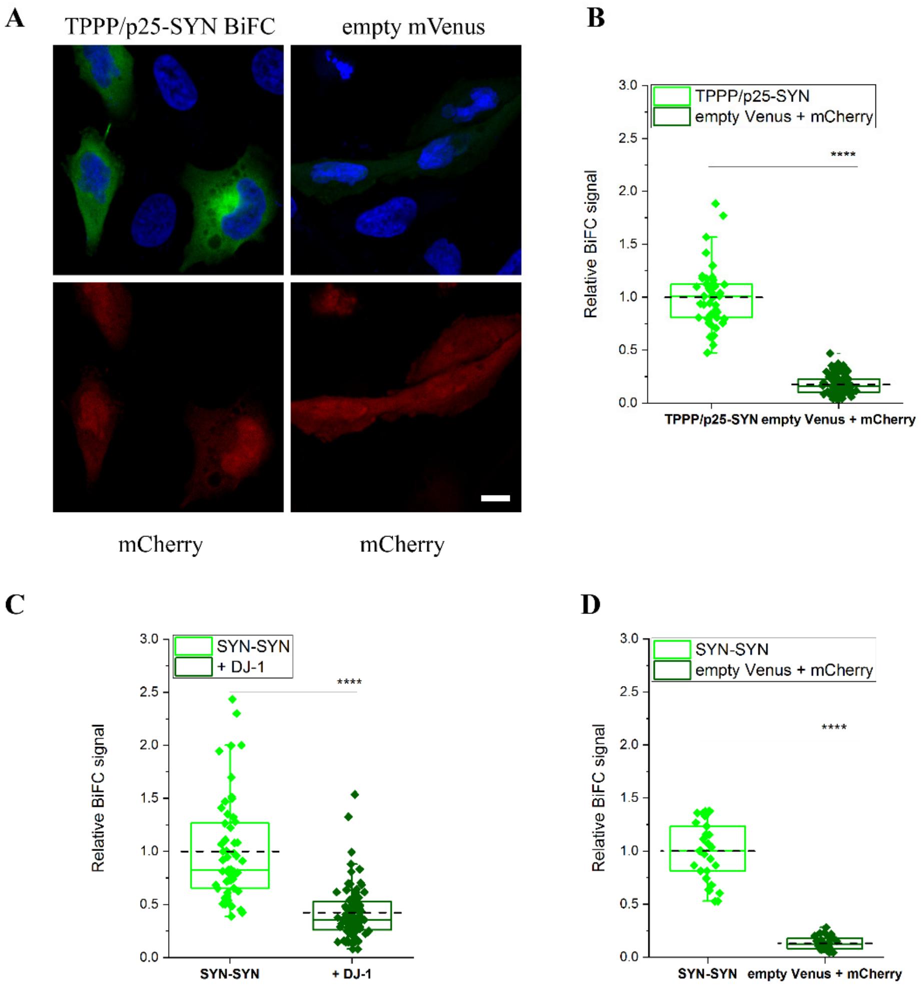

Figure S2. The hetero-association of V^N-SYN with V^C-TPPP/p25 as visualized by BiFC technology. (A) Representative images. V^C and V^N-containing fragments of Venus were conjugated to SYN and TPPP/p25 sequences, respectively, and their plasmids were co-transfected into HeLa cells and also with mCherry. Nuclei, blue (Hoescht 33342). Bar: 5 μm. (B-D) Quantification of the BiFC signal by detecting the individual cell fluorescence: TPPP/p25-SYN versus empty Venus (B), SYN-SYN in mCherry versus mCherry-DJ-1 transfected cells (C), and SYN-SYN versus empty Venus (D). (B-C) Densitometric analysis was carried out as described in the Materials and Methods. Box extends from the 25th to 75th percentile with the middle solid green and the dashed black lines representing the median and the mean, respectively. Statistical comparison was performed with one-way ANOVA followed by Tukey’s test.

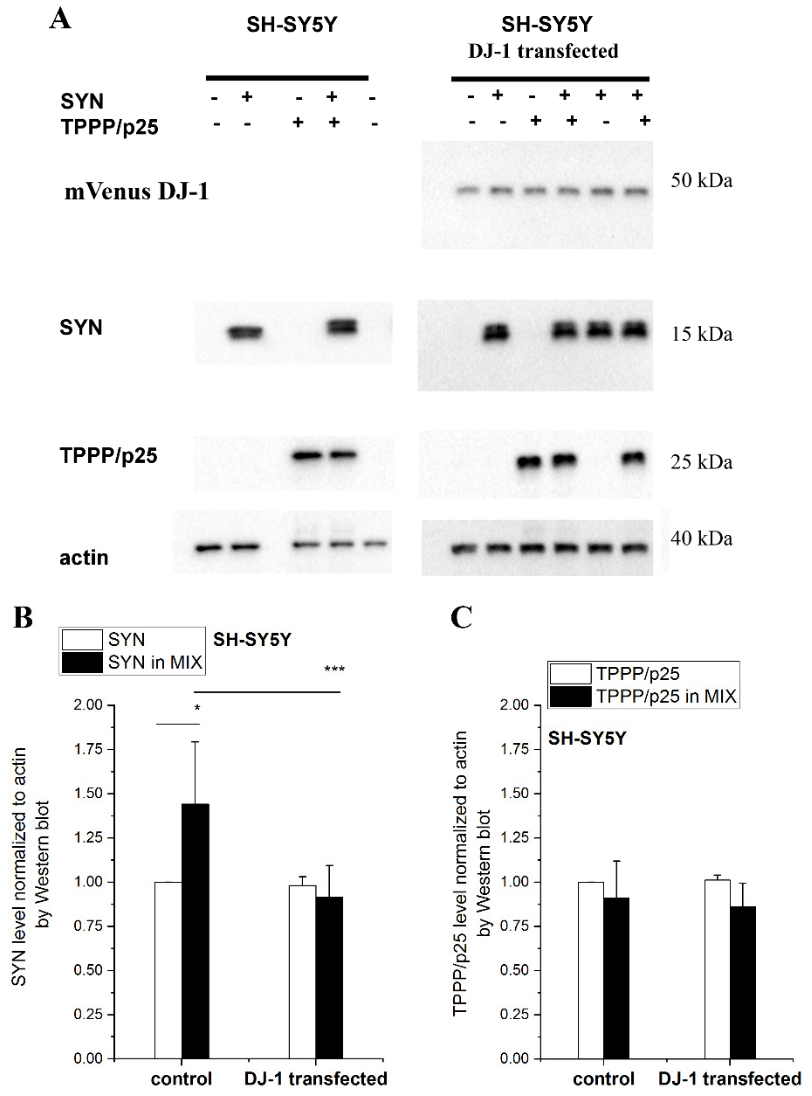

Figure S3. The effect of DJ-1 on the level of the degradation-resistant hallmark proteins in SH-SY5Y cells. TPPP/p25 and/or SYN were taken up from the medium by the cells transfected with DJ-1. (A) Representative Western blots detected in the control and DJ-1 transfected cells. Actin as a loading control is also shown. (B) Proteins levels of SYN and TPPP/p25 quantified by Western blot. Data are normalized with respect to the added hallmark protein alone (SYN or TPPP/p25), and presented as the mean ± SD of at least 3 independent experiments. Statistical comparisons were performed with Kruskal-Wallis non-parametric test (***$p < 0.001$ and *$p < 0.05$ compared to the control or as the lines indicate).

Table S1. Quantitative analyses of pixel intensities and co-localization analysis (Pearson's correlation coefficient; R) of the green (BiFC or mVenus signal) and the red (tubulin) channels.

Sample	Mean intensity of green (arbitrary unit)	Mean intensity of red (arbitrary unit)	Pearson's correlation coefficient, R
BiFC TPPP/p25-DJ-1	49.22 ± 23.51	67.92 ± 40.12	0.79 ± 0.08
BiFC SYN-DJ-1	61.78 ± 25.78	51.20 ± 17.63	0.02 ± 0.08
mVenus DJ-1	28.26 ± 23.19	29.42 ± 20.59	0.29 ± 0.07
BiFC SYN-SYN	32.77 ± 15.29	40.24 ± 15.74	0.08 ± 0.09
BiFC TPPP/p25-SYN	59.02 ± 16.79	73.55 ± 21.73	0.36 ± 0.13

The values of Pearson's coefficient (R) and the mean intensity were determined by the LSM Zen 2010 B SP1 software, similarly as described in [1]. Data are presented as the mean ± SD.

Supplementary Reference

1. Fouani, L.; Huang, M.L.H.; Cole, L.; Jansson, P.J.; Kovacevic, Z.; Richardson, D.R. During mitosis ZEB1 "switches" from being a chromatin-bound epithelial gene repressor, to become a microtubule-associated protein. *Biochim Biophys Acta Mol Cell Res.* **2020**, *1867*, 118673, doi: 10.1016/j.bbamcr.2020.118673.
